# Epistemic discourses concerning the competence developed in a norwegian master’s degree program in health science

**DOI:** 10.1007/s10459-023-10253-8

**Published:** 2023-06-12

**Authors:** Margareth Kristoffersen, Bjørg Frøysland Oftedal

**Affiliations:** 1 Department of Care and Ethics, Faculty of Health Sciences, University of Stavanger, Stavanger, Norway; 2 Department of Quality and Health Technology, Faculty of Health Sciences, University of Stavanger, Stavanger, Norway

**Keywords:** Competence, Critical thinking, Discourse, Health professional, Master’s in health science, Scientific thinking

## Abstract

It has been claimed that various discourses related to competence influence higher education, but there is limited understanding of the discourses underlying competence development. The specific aim of this study was to explore epistemic discourses concerning the development of competence of health professionals with a master’s degree in health science. Accordingly, the study was qualitative and adopted discourse analysis. Twelve participants, all of whom were Norwegian health professionals aged between 29 and 49 years, participated in this study. Four participants were in the final stage of study for their master’s degree with three months left before completion, four had completed their degree two weeks before their participation, and four had been working for one year after the completion of their degree. Data were collected in three group interviews. Three epistemic discourses were revealed: (1) a critical thinking competencies discourse, (2) a scientific thinking competencies discourse, and (3) a competence-in-use discourse. The former two discourses were considered the dominant discourses and indicated that a knowing “that” discourse connected the specialized competence of different health professionals with a wider field of competence. This wider field transcended the boundaries of various health disciplines and represented a novel competence developed through a synergizing process between critical and scientific thinking competencies, which seems to drive continued competence development. A competence-in-use discourse was formed in the process. This discourse can be viewed as a unique outcome that contributes to health professionals’ specialized competence and suggests that a knowing “how” discourse was also an underlying background discourse.

## Introduction

In recent decades, European organizations and governments have increased the demand for highly specialized competence and given more attention to workplace readiness (EHEA, 2005; Clarke, 2018). Consequently, graduate employment has been a salient issue in higher education and represents a shift from discipline-based higher education to occupation-based higher education to provide students with the competence necessary to pursue specific professions (Bergsmann et al., 2015; Ellström, 1997; Mulder, 2014). Therefore, it is particularly interesting to explore competence developed in the educational setting of a master’s degree program in health science where the students who attend and influence this program have bachelor’s degrees in different health disciplines.

In this study, competence development is explored in the context of a Norwegian two-year full-time discipline-based master’s degree program in health science (120 credits according to the EU 2015). This master’s degree program highlights perspectives drawn from different disciplines, including human sciences, social sciences, and health sciences. It corresponds primarily to national health services and references international health services. The program also includes the perspectives of different partners, such as health professionals, users and health services. To initiate advanced knowledge development in the field of health services, the curriculum is research-oriented and prepares the student to perform independent scientific work. Its content includes two main parts, one dedicated to the theoretical concept of health and health promotion and the other to moral philosophy and the philosophy of science (each 10 credits). Moreover, the content includes courses in methodology and scientific methods, both qualitative and quantitative (20 credits). Several elective courses relevant to health science are offered, such as health technology in clinical practice, user involvement and person-centered care in the health service, and patient safety in theory and practice (each 10 credits). To stimulate students to explore beyond what is known and engage in knowledge construction, their work on a master’s thesis (50 credits) in the form of monographs or articles begins in the second semester. After graduating, students are qualified to engage in evidence-based practice and improve health services. They can apply for admission to a PhD program or work within a wide range of academic and management positions in health services.

To strengthen the quality of professionals’ competence, universities and university colleges play a pivotal role in its development. Competence, which is often related to the formal outcome of an assessment process, is obtained when a student has achieved certain learning outcomes according to established standards (EHEA, 2005; Bergsmann et al., 2015; Tremblay et al., 2012). However, Vygotsky (1978) argues that competence is always in the making, implying that it is not given in a particular setting and that both knowledge and skills are necessary competencies in the process of competence development. That is, competence consists of various competencies. The researchers ten Cate and Schumacher argue that competencies are “specific components of overall competence, suitable for specific tasks” (2022, p.492). Competencies are therefore often perceived as a diverse set of qualifications that support the implementation of various skills (Cowan et al., 2005), including both technical and nontechnical skills (Lane, 2010) and, often, behavioral qualities or personal attitudes and values (Epstein & Hundert, 2002; Talbot, 2004). Competencies are not immediately apparent as being suitable for specific tasks. Rather, they are abilities that “do not exist outside individuals” and “need a context to make them visible” (ten Cate & Schumacher, 2022, p.492). The boundaries between competence and competencies are nonetheless blurred (Cowan et al., 2005; Edwards & Daniels, 2012). Moreover, the notion of competence can be associated with and refers to practices that are connected with one another in a given setting in which knowledge is constructed and warranted (Cetina, 2007; Marsick et al., 2014).

There have been several attempts in the literature to understand professionals’ development of competence. For example, Benner (1984) described how registered nurses develop competence in stages from novice to expert, and Munangatire and McInerney (2021) explored nursing students’ conceptions of competence in terms of nursing practice. Kristoffersen and Oftedal (2020a) also studied critical aspects in the development of PhD candidates’ professionally relevant, practice-near research competence in the fields of health, welfare and education. Students’ competence development and achievement are often considered interactional and interpretative, implying that discourses are embedded in a setting. Discourses are “where meaning comes from” (Hall, 2001, p.73) and are available through discussions as they focus on describing, negotiating, and justifying preferred versions of social reality in words (Miller & Silverman, 1995). Thus, “all discourses construct positions, from which alone they make sense, become meaningful and have effects,” and they represent “a form of power which makes individual subjects” (Hall, 2001, p. 80). Discourses can therefore be used to highlight the positions that an individual could occupy with respect to the diversity of discourses in a particular context (Foucault, 1972). Hence, it has been claimed that various discourses related to competence influence higher education (Mulder et al., 2007).

### Theoretical perspective

In this study, two dominant epistemic discourses that have been viewed as part of higher education serve as the basis for analyzing health professionals’ epistemic discourses concerning competence development. The first is a knowing “how” discourse, which refers to knowledge in the form of knowing how to do something that is necessary to achieve the goods of a practice (Skirbekk & Gilje, 1996). Mulder et al. (2007) claim that a knowing “how” discourse informs competence influenced by a behavioristic approach. This approach underscores competences that are considered salient to the competencies for engaging in successful and effective work. It is thus frequently described in terms of procedural knowledge and skills (Arnold et al., 1999) and emphasizes what a professional has, the competence necessary to perform work, also known as “competence-in-use” (Ellström & Kock, 2009, p.7).

The second dominant discourse concerning competence development is a knowing “that” discourse (Mulder et al., 2007). Viewed as a part of the higher educational field, this discourse is often distinguished from the knowing “how” discourse (Hyytinen et al., 2014). Knowing “that” refers to substantial knowledge, that is, knowledge regarding universals and the causes of things (Skirbekk & Gilje, 1996; Reeve, 1933). Hence, Mulder et al. (2007) claim that a knowing “that” discourse informs competences that are influenced by a cognitive approach. It stresses the competence that is considered necessary for engaging in critical thinking and requires specific discipline-based knowledge (Hyytinen et al., 2018; Skirbekk & Gilje, 1996). A precursor to the tradition of modern critical thinking, the American philosopher and educator John Dewey, defines critical thinking as “active, persistent, and careful consideration of any belief or supposed form of knowledge in the light of the grounds that support it, and the further conclusions to which it tends” (1910, p. 9). Accordingly, critical thinking implies the theoretical elaboration of a focal issue. Elaborating an issue theoretically entails identifying and assessing relevant scientific data as well as using scientific ideas to interpret them and reach well-reasoned conclusions, which includes weighing those conclusions against relevant scientific criteria (Hyytinen et al., 2014). In addition, theory must be translated into practice and communicated effectively to others in relation to scientific issues (Hyytinen et al., 2014). There is, however, a dispute regarding whether critical thinking is general and shared across all disciplines or discipline specific. Hyytinen et al. (2018) argue that critical thinking involves both. More precisely, critical thinking is viewed as a foundation of scientific thinking (Holma & Hyytinen, 2015; Reeve, 1933), which again requires a mature epistemic belief (Hofer, 2002). A mature belief recognizes the epistemological foundations of science that identify knowledge as uncertain and constructed by human beings based on the methods used in knowledge construction. Both critical and scientific thinking are connected to generic thinking, such as communication, problem solving and conflict resolution, implying that generic thinking is relevant across a variety of professional practices (Mulder, 2014). Generic thinking is also said to include reflexivity (Archer, 2012), which involves making something one’s own by identifying a particular concern, deliberating over the potential courses of action, deciding what is feasible in a situation, and, ultimately, deciding on a way forward.

The current study seeks to unpack epistemic discourses concerning competence developed in a master’s degree program in health science. It accomplishes this task by exploring discourses that are addressed and referenced in health professionals’ discussions and the ways in which they position themselves and organize their discussions, which in turn form the context for any subsequent discourse. Empirical research on this issue is limited, and there is little information regarding discourses underlying competence development. Moreover, exploring epistemic discourses is critical because it allows an understanding of the unique or privileged access to knowledge and skills that a master’s degree program in health science can add to health professionals’ competence with regard to shaping professional practice. This unique contribution may reveal how epistemic discourses concerning competence development transcend the boundaries of different health disciplines and connect health professionals’ specialized competence with a wider field of competence. Accordingly, the specific aim of this study was to explore epistemic discourses concerning the development of competence of health professionals with a master’s degree in health science.

## Methodology

### Study design

A qualitative design based on discourse analysis was employed. This approach extends from the ethnomethodological discourse analytical tradition (Miller & Silverman, 1995; Potter & Wetherell, 2001), which includes being “concerned with the meanings that events and experiences hold for social actors,” such as the human process of meaning-making or language in use (Wetherell et al., 2001, p.1). According to Wetherell et al., such an approach provides “a perspective on social interaction and an approach to knowledge construction across history, societies and cultures” (2001, p.1). The approach focuses on individual discursivity rather than on collective discourse. It is therefore suited to both the process of competence development and discussions regarding the development associated with earning a master’s in health science, which are considered interactional and interpretative. This approach makes it possible to study the issues that are discussed and positions that are taken in the focal context on a moment-by-moment basis in and through these interactions (Wetherell, 2001). This approach also includes both a bottom-up and top-down focus, that is, both the participants’ point of view and an institutional perspective (Miller & Silverman, 1995).

#### Participants

The sampling, which was conducted by employing convenience procedures (Polit & Beck, 2017), had the following inclusion criteria: students and graduates who had attended a two-year regular master’s degree program in health science in three different years (2017, 2018 and 2019), with a population of approximately 20–25 students each year (for a total of approximately 60–70 students). An administrative coordinator of the faculty recruited the participants. Twelve participants, eleven female and one male, between the ages of 29 and 49 years (median 38 years) agreed to participate in the study. This gender split was representative of the students in and graduates of the focal master’s degree program. The participants had heterogeneous health professional backgrounds and represented six health professions. The participants also had wide-ranging professional work experience ranging from one and a half to 24 years (median 18 years) in public health service, with two participants in private health services. One participant had work experience as a formal leader. Six participants had previously graduated and were at a postgraduate level. Four participants had three months left before graduation, four had completed their master’s degree two weeks before participation, and four had worked for one year after the completion of their master’s degree. These graduates had experienced the process of transition from master’s students to employees and could therefore assess the utility of the competence they had achieved in their degree program.

#### Data collection

Group interviews were used to collect data (Fontana & Frey, 2005; Malterud, 2017). This approach was adopted to resemble everyday conversations as closely as possible and to capture the participants’ responses in the context of face-to-face interactions (Kamberelis & Dimitriadis, 2005). Each group consisted of four participants who met once for one and a half hours at the university in 2020 (two groups) and 2021 (one group). To start each discussion, one researcher (MK) asked an initial question, after which the other researcher (BFO) focused on the follow-up questions.

The first question asked the group to discuss the competence that they had developed as part of their master’s degree program in health science. To assess the ways in which the participants spoke about the process of competence development, they were encouraged to discuss their competence in health science freely and at length based on the following questions: What does this competence mean for you? What are the properties or features associated with competence? To encourage the participants to expand on their initial statements, more specific questions were also posed, such as, “How has your competence developed since you started the master’s degree program in health science?,” “What are the more specific changes in your competence?,” and “What are the implications of your new competence for your employability, that is, workplace readiness and the alignment between curricula and workplace needs?” By asking these open questions, the researchers avoided leading the participants’ answers. All participants actively engaged in group discussions. The participants supported each other, and their reflections were further developed by challenging each other and introducing opportunities for multiple meanings, which allowed the discussions to serve as consolidations of perspectives and interactions. The group discussions were audiotaped and then carefully transcribed verbatim.

#### Ethical considerations

The Norwegian Centre for Research Data approved the study (no. 440314). Verbal and written information regarding the study was given to the master students enrolled in three different years. Individuals who wished to participate in the study were asked to send an e-mail to one of the researchers, and their written consent was obtained prior to their participation. Confidentiality was guaranteed by removing all identifying information regarding individual participants, and they were notified of their right to withdraw.

#### Analysis

The analysis of the interview material entailed a careful notation of the meaning-making processes, that is, the participants’ discursive actions, debates and language in use as well as the processes of interaction between the participants and the researchers conducting the interviews (Miller & Silverman, 1995; Potter & Wetherell, 2001). To engage with the participants’ debates and create a preliminary description, the researchers listened to the discussions and read and reread the interview transcripts, carefully examining the debates that occurred during the discussions regarding the emergence and development of competence associated with a master’s degree in health science. Reflective questions were posed to elicit descriptions of the reasons underlying these debates or utterances. What did the utterances do or accomplish? What was at stake? What was communicated about the wider discursive debates that influenced what was available to be said? The analysis focused on dominant utterances or debates as well as shared senses and variations, which depended on the contexts of use and the positions that the participants took during the discussions.

Both researchers were involved in the analysis and coordinated several informal meetings to ensure that they reflected on, described, and examined the descriptions of the text and identified the corresponding discourses. The preliminary analysis involved the tentative clustering of extracts from each transcript by one of the researchers (MK). These extracts were then read again to condense and transform the data by naming and describing the dominant epistemic discourses and identifying any variation in the descriptions. In the interpretation phase, relevant methodological literature was reviewed in relation to the descriptions, which were read and reread to reflect on various parts of the text and to interpret and thematize the constructed version of the epistemic discourses regarding competence development. Internal verification was appropriately and continuously conducted to maintain an open mind, to be sensitive to nuances in the text and to focus on the ways in which the discourses highlighted what the participants discussed, which are described as the results.

## Results

The discourse analysis revealed three epistemic discourses: (1) a critical thinking competencies discourse, (2) a scientific thinking competencies discourse, and (3) a competence-in-use discourse.

### Critical thinking competencies discourse

The discourse highlighted the participants’ active awareness of critical thinking competencies. Their awareness of the development of such competencies was constituted in and through the group interactions: “I didn’t think I had developed as a critical thinker, but when you emphasize in this discussion that our critical thinking competencies have been developed, I do understand that it’s true” (D1.2). The development of critical thinking competencies typically passed through stages from a lack of awareness to clarity in terms of how the participants’ awareness of these competencies was fostered. Notably, disciplinary knowledge was viewed as a basis for the participants’ critical thinking:

I can argue professionally, based on my discipline knowledge, but my critical thinking skills were not extraordinarily strong at the bachelor’s level. Thinking critically for yourself, no, this was not a strong competence before. Thus, my critical thinking skills were developed and strengthened after I attended the master’s program in health science (D1.4).

This discourse identified the participants’ agreement regarding the fact that their critical thinking competencies had been developed. They supported each other’s claims. None of the participants opposed the statement that these competencies were newly developed. The participants claimed that critical thinking was not a strong competence for them before the program and that it had been achieved through their opportunity to complete the master’s program in health science. At a level of education higher than a bachelor’s degree, the critical thinking competencies of the participants were related to a broad knowledge base, which they identified as “an achievement of repeatedly applying knowledge in health science” (D1.3). In fact, these competencies contributed to an improvement in the health professionals’ discipline knowledge:

Critical thinking competencies are particularly useful and represent a competence that adds to the former specific health professional competence. Critical thinking competencies are attractive as these skills revolve around argumentations concerning the rationale for why we do things, and we have the capability to argue why we do what we do (D1.1).

This quotation demonstrates that critical thinking competencies were integrated into the participants’ disciplinary knowledge base and that they formed a wider framework of horizons for their thoughts and actions. These competencies allowed the participants to adopt alternative perspectives on relevant health issues, including the skills to elaborate on these issues and to explore them theoretically by identifying and assessing relevant scientific data to draw well-reasoned conclusions. These competencies were said to be useful. Health issues were thus better understood, and the proper course of action, as a health professional, was considered against the backdrop of relevant scientific criteria. These competencies enabled the participants to listen to what was said in a discussion and why it was said more effectively than they could previously: “I listen to what is referred to—are the references included in what is said and such—focusing on something that is interesting” (D2.1). These competencies also involved posing questions related to these issues, investigating the reasons underlying a conclusion and asking generic critical questions, such as “Is what is being said really true?,” “How has the knowledge been developed?,” and “Is the background of what is being said based on evidence?” Accordingly, it was noted that “there is never one answer to questions, there is seldom one reference, and it is possible to perform considerations ‘on the road’” (D1.2). The competencies associated with critical thinking strengthen individuals’ independence with regard to assessing health issues critically, grounding their standpoints in a knowledge base and providing arguments for supporting these standpoints. Critical thinking changed the health professionals’ professional argumentation and attitudes. These competencies both legitimated their opinions and made it easier for them to communicate with others in a wider professional and social community, whether in the classroom or in the workplace.

### Scientific thinking competencies discourse

The discourse revealed that the knowledge and skills associated with methodology and scientific methods were pivotal in competence development. Scientific thinking competencies were described “as important knowledge and skills that are instructive with regard to our research” (D2.4). This quotation implies that the competencies were related to knowledge construction and the methods of conducting research. Study toward a master’s degree in health science was characterized as “research heavy” (D3.1). In the discussions, scientific thinking competencies were described as the “most heavy knowledge and skills that studying for the master’s has brought us and that we have developed during studying for the master’s” (D1.1).

Scientific thinking competencies were claimed to represent the development of competence: “Methodology and scientific methods are new knowledge and skills; it’s a change, and it’s interesting, yes!” (D1.2). This discourse indicated that participants lacked the knowledge and skills related to research before attending their master’s degree program in health science, implying that these health professionals were not previously trained in the use of scientific methods. To a large degree, methods of finding answers to a research question, including gathering and reviewing information about a topic and analyzing and interpreting the information required to solve a relevant problem, were previously unknown to the participants: “Since scientific methods and research processes are emphasized in the curriculum, it has provided us with important knowledge and skills, resulting in a better understanding of what research is” (D3.1). The competencies associated with scientific thinking involved performing literature searches and reading research to complete their master’s project and evaluate the scientific quality of research conducted by themselves and other researchers. This situation strengthened feasible argumentation and promoted the use of references to obtain evidence-based knowledge:

We can perform a literature search and know how to evaluate the quality of the studies we read. When it is easier to read scientific articles, then it is easier to conduct research. I know where to find research, to perform searches in databases and what the scientific criteria are. Thus, the foundation for my words has become better (D1.4).

Some of the health professionals found “the quantitative scientific methods difficult to learn” (D 3.4). Nonetheless, the repetition of methodology and scientific methods during their work on their master’s project improved their scientific thinking competencies:

A repetition of scientific methods has resulted in advanced skills that are relevant to the methods underpinning the master’s thesis, and that’s fine. I have absorbed all that knowledge because it involved skills that were relevant to my master’s project (D3.3).

The discourse highlighted how scientific thinking competencies based on the philosophy of science provided a deeper understanding of scientific thinking: “We have learned how to reflect as researchers; this was learned from the philosophy of science and its link to scientific methods, with implications for conducting research” (D2.1). This quotation demonstrates that the philosophy of science encouraged an understanding of the foundation of research and offered mature insights into the ways of acquiring knowledge and developing the science of human beings’ experiences. A deeper understanding therefore emerged from the ways in which the construction of knowledge, based on the philosophy of science, provides a rationale for research design, the reasons why knowledge is viewed as constructed by human beings, and the methods used in research.

### Competence-in-use discourse

A competence-in-use discourse identified health professionals’ competence in future work settings. However, the group discussions demonstrated that the participants positioned themselves differently in this context.

After completing or nearly completing their master’s degree, the participants had obtained the critical and scientific thinking competencies that are relevant to working as a researcher. Some health professionals identified themselves as potential researchers who wanted to conduct research in their future careers:

I’m qualified to work with research, meaning to plan and conduct research, and I’m also prepared to develop myself further in that direction. I’ve been qualified at an advanced level through the master’s degree program in health science (D2.3).

During their work on their master’s project, the participants learned how to practice scientific methods through real work in academic life. As part of this work, some health professionals also acted as co-researchers and were engaged in the research process associated with a larger research project. This collaboration with experienced researchers motivated the participants to continue their research in the context of academia.

This discourse also identified the participants’ competence with regard to work settings outside academia. This competence was considered important in relation to improving everyday professional practice. In the discussion, the following statement was made:

With our skills as a master in health science, we can do a decent job and have an influence on people. I can find relevant research results about health issues, read and evaluate research results and am capable of writing scientific text about a topic (D1.3).

The discourse thus revealed that the participants had achieved a competence-in-use that had the potential to affect their health professions in the future. It was noted that one “duty is to read scientific articles in order to stay professionally updated, but before obtaining my master’s in health science, this was something I rarely did in my professional practice” (D1.2). Moreover, conducting research within health services was possible as the critical and scientific thinking competencies that the participants had obtained offered them the competence necessary to write a project application and conduct research in structured and systematic ways:

In my health professional practice, we write project applications, and my knowledge and skills have given me the competence necessary to do this work. I’m able to formulate an aim and write an outline of a literature background and include relevant actions. It is a part of a project’s trustworthiness that I do the job properly by demonstrating respect for research results and academic writing (D2.3).

In addition, the discourse identified how the participants’ competence-in-use allowed them to collaborate with researchers in health service research. On some occasions, the health professionals were invited to participate in ongoing research projects. These invitations from researchers were no longer viewed as frightening. The competence achieved during the participants’ work on their master’s projects was therefore useful in their discussions with researchers:

As a health professional working in the health service, I collaborate with a PhD candidate and have self-confidence connected to what I can contribute. I don’t consider research work as difficult as before. By completing my master’s thesis, I gained experience in research and a better understanding of what a PhD project involves. I’m skilled and can consider what a candidate is doing and ask critical questions. I want to obtain a PhD candidate’s arguments concerning choices related to data collection within the health services (D2.3).

The discourse nonetheless emphasized to varying extents the participants’ confidence with regard to their transition from master’s students to individuals with a master’s degree in health science who could utilize critical and scientific thinking competencies. Although a competence-in-use discourse was developed, one issue that emerged was that the health professionals were unsure how they could use their critical and scientific competencies in their future careers: “I have obtained insight into research and how to do research, and I have done research, but I am not experienced and not sure how to use my skills in my future career” (D3.3). This insecurity was perceived as a particular challenge for participants who did not plan to become researchers within academia after completing their master’s degree. The ability to confidently employ the competence related to work settings in a scenario far from the original context of academia was not considered easy. Furthermore, the utility of the development and achievement of this competence was viewed as more tenuously related to the use of critical and scientific competencies in a work context outside academia.

## Discussion

This study employed discourse analysis to explore epistemic discourses concerning the competence developed in a master’s degree program in health science. A critical thinking competencies discourse and a scientific thinking competencies discourse were identified as dominant epistemic discourses in competence development and achievement. These two discourses, which were addressed and referenced in the focal health professionals’ discussions, indicate that knowing “that” is an underlying discourse. Emphasizing a knowing “that” discourse as a fundamental discourse underlying competence development implies that this discourse is relevant in this master’s degree program and that this program primarily focuses on this discourse. In higher education, a knowing “that” discourse incorporates the acquisition of critical thinking and includes both scientific thinking and generic thinking (Mulder et al., 2007; Mulder, 2014; Hyytinen et al., 2018). Moreover, this fundamental background discourse, that is, a knowing “that” discourse, connects the specialized competence of different health professionals with a wider field of competence that transcends the boundaries of various health disciplines. This wider field of competence represents a novel competence that was said to be lacking when health professionals initially commenced their studies toward their master’s degree. At this stage, it is therefore relevant to highlight the synergy of critical thinking and scientific thinking competencies discourses and that this synergy seems to drive health professionals’ continued competence development. Hence, the competence formed by this synergy can be considered a unique outcome that adds to health professionals’ specialized competence, and the epistemic discourse that is formed is a competence-in-use discourse.

The group discussions showed that the health professionals had developed competence in critical thinking, including the competency to adopt alternative perspectives on relevant health issues. In this context, discipline-based skills, such as a professional knowledge base, are considered necessary to engage in critical thinking (Brante, 2013, 2014; Hyytinen et al., 2018). Critical thinking refers to substantial knowledge regarding universals and the causes of things (Skirbekk & Gilje, 1996; Mulder, 2014; Reeve, 1933). Such thinking focuses on the theoretical elaboration of the focal issues, which implies a careful consideration of knowledge in light of the grounds that support it (Dewey, 1910) as well as identifying and assessing the relevant scientific data and reaching well-reasoned conclusions (Hyytinen et al., 2014). In addition, this discourse identifies the health professionals as possessing scientific thinking competencies and addresses their development of knowledge and skills in terms of methodology and scientific methods. These competencies are viewed as essential in allowing these professionals to generate new knowledge, complete their master’s projects, read research literature, improve their academic writing and learn how to employ scientific methods in work settings. The impact of critical and scientific competencies, therefore, does not indicate that the different health disciplines are irrelevant but rather the opposite. Disciplinary knowledge is perceived as a prerequisite for critical and scientific thinking. In this context, previous research has stated that achieving a specific disciplinary knowledge base at the bachelor’s level is helpful, even when conducting research at the PhD level (Kristoffersen & Oftedal, 2020b). In the above discussions, the health professionals emphasized how their developed competence legitimated their opinions. It thus became easier to argue for a rationale related to the proper course of action in professional practice and to communicate with other professionals in a wider professional community. Moreover, this result is in line with previous research that claims that both critical and scientific thinking are connected to generic thinking since communication and problem solving are relevant across different health discipline fields (Mulder, 2014). Because generic thinking can include reflexivity (Archer, 2012), it entails making something one’s own and helps individuals determine what might be feasible in a given situation. Accordingly, generic thinking includes arguing for a certain way of understanding an issue and how to respond to it. Thus, this study indicated that the knowing “that” discourse was the most dominant fundamental background discourse in the focal health professionals’ discussions. However, this study does not indicate that the knowing “how” discourse has no relevance whatsoever.

The group discussions revealed that the health professionals positioned themselves with a competence-in-use discourse. This epistemic discourse indicates that a knowing “how” discourse is an underlying background discourse pertaining to competence development. Although a knowing “how” discourse is another dominant discourse in higher education, particularly in professional education (Mulder, 2014), a competence-in-use discourse seems to be a less dominant discourse in the context of the focal master’s degree program. This discourse nonetheless identifies the relevance of that competence for shaping professional practice. It thus demonstrates how the outcome of health professionals’ competence, which is developed at the master’s level, has the potential to be applied in work settings in both health services and academia. In other words, it shows how critical and scientific thinking are connected to generic thinking (Mulder, 2014) and hence how generic thinking, together with discipline-based competencies, constitutes graduate employability. Previous research has noted that societal changes and constant changes in work settings cause the demand for the development of competence-in-use to increase, and the employability of graduates is related to the competences that they bring into their workplace (Purcell et al., 2013; Meld. St. 16. 2020–2021). Competence-in-use relates to competencies that allow professionals to meet the challenges they encounter within society (Ellström & Kock, 2009; Tremblay et al., 2012, 2013). This competence thus shapes professional practice with respect to the application of evidence-based knowledge in work settings, including new or unfamiliar settings (Brennan et al., 2004; Cochrane and Williams, 2010). Notably, application is an activity that often requires competence development in terms of how to implement evidence-based knowledge into health practice (Schultes et al, 2021). Nevertheless, the synergy between critical and scientific thinking competency discourses seems to represent a unique outcome that contributes to health professionals’ specialized competence.

More critically, the epistemic discourse concerning competence-in-use reveals that health professionals position themselves differently in future work settings. The acquisition of confidence when pursuing a master’s degree in health science using one’s competencies in critical and scientific thinking is not necessarily considered easy. To varying extents, this discourse provides health professionals with confidence in constructing knowledge and performing basic research in their future careers. This interesting result supports the claim that competence must often be acted upon in relation to unique individuals (Havnes & Smeby, 2014). Competencies are abilities that depend on individuals, their behavioral qualities, personal attitudes and values, and a context to be visible (Epstein & Hundert, 2002; Talbot, 2004; ten Cate & Schumacher, 2022). Alternatively, as suggested by Hall (2001), all discourses construct positions; thus, the above result indicates how a competence-in-use discourse has constructed the focal health professionals’ position and represents a form of power that renders individuals differently related to their future work settings. For the health professionals who position themselves as unsure and, in discussions of the way forward, draw attention to the benefit of their novel competence in the long term, this is a troubling position. This position might be accentuated by the fact that the novel competence does not provide the competence necessary to pursue a specific health profession. Additionally, the discourse concerning competence-in-use is interesting with regard to the competence required to contribute to knowledge construction and start work on a PhD project. Research has found that health professionals experience a loss of prestige in terms of competence when they become PhD candidates (Kristoffersen & Oftedal, 2020a). This loss can be a challenge, and the large gaps in competence that must be bridged when health professionals voluntarily leave their professional positions to undertake PhD studies are even more troubling. Hence, this study indicates that the competence associated with a master’s degree in health science, which is based on a fundamental knowing “that” discourse, may reduce this loss of prestige. Because this competence connects health professionals’ specialized competence with a wider field of competence, it transcends the boundaries of different health discipline fields and may facilitate the achievement of the autonomy necessary to earn a PhD and achieve future positions in both health service and academia.

### Strengths and limitations

This study was based on discourse analysis, which is considered suitable for accomplishing the aims of this research. The participants had everyday experiences relevant to the research topic. They had been involved in the development of competence during their studies for a master’s degree in health science. To establish an appropriate environment for discussion, the groups consisted of fellow degree students, the group sizes were small, and the participants had established relationships with one another prior to their discussions, which were held in study-life conditions at a university in Norway.

The chosen data collection method was therefore suitable for deriving data concerning the development of competence from discussions involving the participants’ daily language. The discussions from which the data were drawn took place in a relational manner as the participants engaged in discussions with each other. Multiple discourses were thus employed, including arguments intended to elaborate discussions. Data collection concluded after three groups had been interviewed and the researchers no longer obtained substantial new information. However, it is conceivable that participants without work experience as health professionals or participant groups with several men may have discussed issues other than those mentioned by the participants in this study. Nevertheless, although these results may not be representative of all master’s degree programs related to health science, the study identified certain epistemic discourses that may be relevant to similar theoretical master’s degree programs.

The researchers are aware that multiple perspectives are legitimate and that discussions are open to multiple readings, including their positions regarding questions and follow-up questions. This study did not rest upon a specific proposition as the research approach was inductive; thus, the analysis was not conducted in a deductive way. Nonetheless, the study did not focus on the depth of the competencies discussed or how advanced the participants’ development seemed to be. The results may therefore be considered somewhat “noncritical,” implying that there may be value in the inclusion of differing perspectives from participants beyond the one location where these results were found. For instance, any challenges to the participants’ statements in terms of their critical and scientific thinking competencies or exploration of the aspects of the multidisciplinary group were not elaborated in the group discussions. This could have been interesting given that each of the study’s participants had entered a new discursive space with distinct disciplinary knowledge. Furthermore, the results did not indicate whether the participants had engaged in postgraduate study without critical and scientific thinking competencies developed during their undergraduate studies. However, even though multiple discourses were employed, they did not conflict. The participants may have been less open because two researchers were present and represented a certain kind of expertise as researchers and lecturers. One researcher had met some of the participants as a lecturer two to eight times during one of their master’s courses, while the other researcher had not met the participants or been involved in the master’s degree program. One researcher had also supervised one participant’s work on the master’s thesis, but this participant graduated before participating in this study. This situation may have created distance between the researchers and the participants. However, the invitation to participate was extended by the administrative coordinator, and the students and graduates who consented to participate contacted the researchers via the internet.

### Implications for educational practice

One responsibility associated with higher education is to emphasize an educational strategy that makes connections and enhances understanding of the synergy between critical and scientific thinking competencies as well as the ways in which the impact of this synergy is linked to competence-in-use. This responsibility is relevant as highly competent graduates at the master’s level can participate in the construction of knowledge and the implementation of scientific knowledge with the aim of meeting the needs of society, health services and academia (Numminen et al., 2019). This strategy implies a focus on the interaction between health professionals’ disciplinary backgrounds and the competence they achieve at the master’s level. Promoting discussions of different knowledge discourses can thus advance professionals’ competence as master’s graduates in health science. Moreover, the relevant educational strategy suggests that a curriculum at the master’s level should constructively emphasize discourses of competence, including the ways in which such competence relates to the application of knowing “that” and knowing “how” thinking. Critical and scientific thinking competencies can be developed in seminars and must be promoted systematically during the course of study (Hyytinen et al., 2018). The implementation of such an educational strategy can allow individual master’s students to gain the trust necessary to act independently as health professionals with advanced competence. This should be advocated because this strategy focuses on the promotion of a dedicated discourse intended to facilitate the transition from health professionals to master’s graduates in health science. Explicitly verbalizing one’s competence can promote success in studying for a master’s degree in health science. This goal might be one toward which health professionals can aspire to strengthen their employability and thus their competence in coping with changing work settings.

## Conclusion

The results of this study’s exploration of the epistemic discourses of Norwegian health professionals concerning the competence they developed in a master’s degree program in health science indicate the dominance of critical thinking and scientific thinking competency discourses. These two epistemic discourses indicate that a knowing “that” discourse is a fundamental background discourse for health professionals’ competence development. This novel competence transcends the boundaries of different health disciplines and connects health professionals’ competence with a wider field of competence. It is developed through a process in which critical and scientific thinking competencies are synergized, which seems to drive continued competence development. A competence-in-use discourse is formed by that synergy and can be identified as a unique outcome that contributes to health professionals’ specialized competence. Thus, while knowing “that” thinking is emphasized, knowing “how” thinking is not far below the surface and emerges in the less dominant competence-in-use discourse.

Health professionals with a bachelor’s degree often go on to further study, and the direction of their postgraduate education might be a master’s degree program that allows them to develop competence in critical and scientific thinking. Such competences seem to be worth accentuating in the future. As society and health services persistently change, for health professionals to remain competent, they must be prepared for knowledge construction and the implementation of scientific knowledge in their future careers. This task represents a responsibility or challenge for higher education in the context of a master’s degree program for health professionals. Future research should therefore investigate competence development to prepare health professionals to function at the full scope required by master’s level education. Because this study did not focus on differences in health professionals’ critical thinking competencies, a possible path for future research is to study the differences among different health professionals with regard to such competence-in-use.
